# Analysis of the safety and efficacy of tacrolimus combined with glucocorticoid in the treatment of lupus nephritis

**DOI:** 10.12669/pjms.38.5.5117

**Published:** 2022

**Authors:** Lu Li, Yong Du, Juan Ji, Ying Gao, Xiao-qiang Shi

**Affiliations:** 1 Lu Li, Department of Nephrology, Baoding First Hospital, Baoding 071000, Hebei, P.R. China; 2 Yong Du, Department of Rheumatology and immunology, Affiliated Hospital of Hebei University, Baoding 071000, Hebei, P.R. China; 3 Juan Ji, Department of Nephrology, Affiliated Hospital of Hebei University, Baoding 071000, Hebei, P.R. China; 4 Ying Gao, Department of Urology, Affiliated Hospital of Hebei University, Baoding 071000, Hebei, P.R. China; 5 Xiao-qiang Shi, Department of Urology, Affiliated Hospital of Hebei University, Baoding 071000, Hebei, P.R. China

**Keywords:** Tacrolimus, Glucocorticoids, Lupus nephritis, Treatment

## Abstract

**Objective:**

To evaluate the safety and efficacy of tacrolimus combined with glucocorticoids in the treatment of lupus nephritis.

**Methods:**

A total of 80 patients with lupus nephritis were admitted to the Affiliated Hospital of Hebei University and the First Hospital of Baoding from February 2017 to January 2019 randomly divided into two groups: the experimental group and the control group, with 40 cases in each group. Patients in the experimental group were treated with tacrolimus combined with glucocorticoids, while patients in the control group were treated with cyclophosphamide combined with glucocorticoids for one year. Clinical efficacy and adverse drug reactions were evaluated for all patients after treatment. The changes of CRP, IL-6, 24h urinary protein, serum albumin, serum creatinine, urea nitrogen and other indicators after treatment, as well as the differences in the erythrocyte sedimentation rate (ESR), complement C3, C4, anti-dsDNA antibody positive rate and SLEDAI score and other indicators were also evaluated.

**Results:**

The total efficacy of the experimental group was 92.5%, which was significantly better than the 75% of the control group (p=0.03); The incidence of adverse reactions was 20% in the experimental group and 42.5% in the control group, with a statistically significant difference (p=0.03). After treatment, the levels of CRP, IL-6 and other inflammatory factors in the experimental group were lower than those in the control group, with a statistical significance (p<0.05); The indicators of the experimental group such as 24h urine protein quantification, serum albumin, blood creatinine, and urea nitrogen were improved compared with the control group, with statistically highly significant differences (p<0.001). In addition, ESR, anti-DSDNA antibody positive rate and SLEDAI score were decreased compared with the control group, while complement C3 and C4 levels were significantly increased (p<0.05).

**Conclusion:**

Tacrolimus combined with glucocorticoids is a safe and effective treatment regimen for patients with lupus nephritis, boasting a variety of benefits, such as significant efficacy and fewer adverse reactions. With such a regimen, the level of inflammatory factors can be significantly reduced, renal function indicators can be ameliorated, the ESR, complement C3, C4, anti-dsDNA antibody positive rate and SLEDAI score of the patients can be significantly improved.

## INTRODUCTION

Systemic lupus erythematosus (SLE) is an autoimmune-mediated chronic inflammatory disease characterized by multiple system organ damage and multiple autoantibody production,^1^ with kidney injury being the most common. Lupus nephritis (LN) is the most common organ involvement in systemic lupus erythematosus and a major risk factor for disease progression and death.^2^ Over the past few decades, the pathophyphysiology of LN has become increasingly well understood and treatments for it have improved.^3^ Glucocorticoid combined with cyclophosphamide was the first-line treatment in the previous treatment of this disease, which can effectively improve the renal function of patients with definite effect.^4^ However, the clinical application of cyclophosphamide is sometimes limited due to its side effects such as bone marrow suppression, immune system disorders and infertility.^5^

In recent years, with the continuous in-depth research on the pathogenesis of LN, multi-target immunosuppressive therapy has become a new treatment method.^6^ As a calcineurin inhibitor, tacrolimus can selectively inhibit T lymphocytes, inhibit the production of cytokines, and play a strong immunosuppressive role.^7^ It has been shown in recent years to perform better than cyclophosphamide in the treatment of LN, with better security and fewer adverse reactions.^8^ Based on this, this paper discusses the efficacy of tacrolimus combined with glucocorticoid in the treatment of lupus nephritis..

## METHODS

Eighty patients with lupus nephritis who were admitted to the Affiliated Hospital of Hebei University and the First Hospital of Baoding from February 2017 to January 2019 were randomly divided into two groups: the experimental group and the control group, with 40 cases in each group. Among them, there were seven males and 33 females in the experimental group, aged 30-57 years with an average of 48.06±7.13 years, and nine males and 31 females in the control group, aged 33-60 years with an average of 47.83±9.01 years. No significant difference can be seen in the comparison of general data between the two groups, which was comparable between the two groups (Table-I).

**Table I:** Comparative analysis of general data between the experimental group and the control group (χ¯±S) n=40.

Indicators	Experimental group	Control group	t/χ^2^	P
Age (years old)	48.06±7.13	47.83±9.01	0.13	0.90
Female (%)	33(47.5%)	31(55%)	0.31	0.58
Duration (month)	27.32±9.71	27.95±9.83	0.29	0.77
Severe (%)	7(17.5%)	10(25%)	0.67	0.42
SLEDAI score	14.75±3.43	14.28±3.32	0.63	0.54

P>0.05

### Ethical Approval:

The study was approved by the Institutional Ethics Committee of Affiliated Hospital of Hebei University, and written informed consent was obtained from all participants.

### Inclusion Criteria:

- All patients meeting the diagnostic criteria for SLE;^9^
- Patients aged between 30 and 60 years old, gender is not limited;
- Patients with moderate and severe nephritis conforming to the classification criteria of lupus nephritis;^10^
- All patients with lupus nephritis confirmed by renal biopsy and pathology;
- Patients with active disease, SLEDAI score ≥ 10,^11^ urinary protein quantity ≥ 1.0g/24h;
- Patients who have not recently used other immunosuppressants affecting the study;
- Patients whose family members signed the consent form and were able to cooperate with the study.

### Exclusion Criteria:

- Patients with primary nephropathy or other types of secondary nephropathy;
- Patients with other autoimmune diseases such as dermatomyositis, scleroderma, vasculitis and other autoimmune diseases;
- Patients with metabolic diseases or chronic wasting diseases such as tumors and chronic inflammatory diseases;
- Pregnant or lactating women;
- Patients with infectious diseases such as tuberculosis and hepatitis or with severe and important organ dysfunction such as liver and kidney insufficiency;
- Patients who are allergic, intolerant or have contraindications to the drugs involved in the study.

Both groups were treated with glucocorticoids. The specific regimen was as follows: the initial dose of methylprednisolone tablet was 1.0mg/ (kg/d) in the morning, and the dosage was gradually reduced according to the condition for 4-8 weeks, and 5-20mg/daily was reduced every other week. Subsequently, maintenance therapy was followed with a reduction of 2.5-10mg/d every two weeks for one year. Patients with severe lupus requiring shock therapy were treated with methylprednisolone 0.5-1.0g/d shock therapy for three days, followed by oral administration as described above. Calcium antagonists were used to control blood pressure in hypertensive patients, and the antihypertensive program would be adjusted according to the specific situation.

The experimental group was given tacrolimus therapy.^12^ The specific treatment plan is as follows: Tlimus 0.1mg/(kg·d) was taken two hour after meal twice, and the blood concentration of tlimus was detected 3d after taking the drug. After that, patients were tested once a month to maintain their blood drug concentration at 5-10ug/L, and the therapeutic dose was adjusted according to the blood drug concentration, with a one-year treatment time.^13^ At the same time, the control group was treated with cyclophosphamide, with the specific scheme as follows: Cyclophosphamide with 0.5g/m2 body surface area was administered once a month for six months, and then once every 3 months for one year.^14^ All patients were observed and followed up for one year.

### Observation Indicators:

*Judgment of efficacy*: The clinical efficacy of the two groups after one year of treatment was compared and analyzed: *Complete remission (CR):* No active urine sediment (urinary RBC<10×104ml, no white blood cells and casts); Urine protein quantitative <0.4g/24h, serum albumin ≥35gL, normal SCr, with no extrarenal lupus activity. *Partial remission (PR):* Urinary protein quantification is 0.4-2.0g/24h or decreases by more than 50% of the basic value, serum albumin ≥30g, SCr is normal or the increase does not exceed 15% of the normal range, with no extra renal lupus activity. *Non remission (NR):* Urine protein quantification ≥2.0g/24h and the decrease is less than 50% of the basic value, or the serum albumin is less than 30g/L, or the SCr rises more than 50% of the basic value. Effective rate = (complete remission + partial remission) number of cases/total number of cases × 100%. Inflammatory factor indicators: Peripheral venous blood was collected from all patients before treatment and at basic state from morning on three months after treatment, respectively. The levels of inflammatory factors such as C-reactive protein (CRP) and interleukin-6 (IL-6) were detected by enzyme-linked immunosorption (ELISA).

### Improvement of renal function:

The differences in urinary protein quantification, serum albumin, serum creatinine, urea nitrogen and other indicators between the two groups were recorded and compared before treatment and 24h after treatment for three months. The changes of ESR, complement C3, C4, anti-dsDNA antibody positive rate and SLEDAI score were recorded and compared between the two groups before treatment and 3 months after treatment. The patients’ blood pressure, blood glucose, temperature, blood routine examination, liver function and other indicators were monitored. The incidence of adverse drug reactions such as leucopenia, blood glucose elevation, gastrointestinal reaction, liver function abnormality, infection and so on in the two groups within six months after treatment was compared and analyzed.

### Statistical Analysis:

All the data were statistically analyzed by SPSS 20.0 software, and the measurement data were expressed as (X̄± s). Two independent sample t-test was used for inter-group data analysis, paired t test was used for intra-group data analysis, and c^2^ was adopted for rate comparison. P<0.05 indicates a statistically significant difference.

## RESULTS

The comparative analysis of the efficacy between the two groups is shown in Table-II, indicating that the total effective rate of the experimental group after treatment was 92.5%, which was significantly superior to 75% of the control group, with a statistically significant difference (p=0.03).

**Table II:** Comparative analysis of the efficacy between the two groups (χ¯±S) n=40.

Group	CR	PR	NR	Effective rate
Experimental group	19	18	3	92.5%(37/40)
Control group	16	14	10	75%(30/40)
χ^2^				4.50
P				0.03

P<0.05.

The changes of inflammatory factors in the two groups before and after treatment are shown in Table-III, indicating that there was no significant difference in the levels of inflammatory factors such as CRP and IL-6 between the two groups before treatment (p>0.05). However, the above indicators decreased after treatment compared with before treatment, showing a statistically significant difference (p<0.05). After treatment, the above indicators of the experimental group were significantly lower than those of the control group, with a statistically significant difference (p=0.00).

**Table III:** Comparative analysis of changes in inflammatory factors before and after treatment in the two groups (χ¯±S) n=40.

Group		Before treatment*	After treatmentΔ	t	P
CRP (mg/L)	Experimental groupΔ	87.43±13.67	13.48±4.57	32.45	<0.001
	Control groupΔ	86.80±12.95	18.63±4.81	31.21	<0.001
	t	0.22	4.91		
	p	0.83	<0.001		
IL-6(ng/L)	Experimental groupΔ	17.64±5.61	8.52±2.45	9.42	<0.001
	Control groupΔ	18.02±5.53	13.18±3.21	4.79	<0.001
	t	0.31	7.30		
	p	0.76	<0.001		

* p>0.05, Δp<0.05

Twenty hour after treatment, urinary protein quantitative, serum albumin, serum creatinine, urea nitrogen and other indicators in both groups were improved compared with those before treatment (p=0.00), while the improvement in the study group was more significant than that in the control group, with a statistically significant difference (p<0.001) (Table-IV).

**Table IV:** Comparative analysis of renal function indicators of the two groups before and after treatment (χ¯±S) n=40.

Group		Before treatment*	After treatmentΔ	t	P
24h urine protein quantification (g/24h)	Experimental groupΔ	4.45±1.32	1.14±0.58	14.52	<0.001
	Control groupΔ	4.74±1.61	2.87±0.72	6.71	<0.001
	t	0.82	11.44		
	p	0.38	<0.001		
Albumin (g/L)	Experimental groupΔ	26.58±5.07	45.70±5.79	15.71	<0.001
	Control groupΔ	25.97±5.61	40.83±5.28	12.20	<0.001
	t	0.50	3.93		
	p	0.61	<0.001		
Serum creatinine (umol/L)	Experimental groupΔ	147.52±25.74	77.63±11.82	15.61	<0.001
	Control groupΔ	149.83±27.64	89.71±12.44	12.54	<0.001
	t	0.38	4.45		
	p	0.70	<0.001		
Urea nitrogen (mmol/L)	Experimental groupΔ	14.57±2.06	6.13±1.07	23.00	<0.001
	Control groupΔ	13.97±1.28	9.76±1.58	13.09	<0.001
	t	1.56	12.03		
	p	0.12	<0.001		

* p>0.05, Δp<0.05

After treatment, the ESR, anti-dsDNA antibody positive rate and SLEDAI score of the two groups were lower than those before treatment, and the levels of complement C3 and C4 were higher than those before treatment. The above changes were more obvious in the experimental group than in the control group, with statistically significant differences (Complement C3, C4, SLEDAI score, p<0.001; anti-dsDNA antibody positive rate, p=0.02) (Table-V).

**Table V:** Comparative analysis of the disease change indicators of the two groups before and after treatment (χ¯±S) n=40.

Group		Before treatment*	After treatmentΔ	t/χ^2^	P
ESR (mm/h)	Experimental groupΔ	34.25±8.03	17.53±4.62	11.41	<0.001
	Control groupΔ	33.76±7.82	23.47±5.27	6.90	<0.001
	t	0.28	5.36		
	p	0.78	<0.001		
C3(g/L)	Experimental groupΔ	0.47±0.08	0.78±0.07	30.34	<0.001
	Control groupΔ	0.45±0.03	0.73±0.05	41.22	<0.001
	t	1.48	11.03		
	p	0.14	<0.001		
C4(g/L)	Experimental groupΔ	0.24±0.08	048±0.07	14.28	<0.001
	Control groupΔ	0.25±0.06	0.32±0.06	5.22	<0.001
	t	0.63	10.98		
	p	0.53	<0.001		
Anti-dsDNA antibody (%)	Experimental groupΔ	100%(40)	10%(4/40)	65.45	<0.001
	Control groupΔ	100%(40)	30%(12/40)	43.07	<0.001
	χ^2^	5.00		
	p	0.02		
SLEDAI score	Experimental groupΔ	15.78±3.27	4.76±1.29	19.83	<0.001
	Control groupΔ	15.30±3.06	8.65±2.40	10.81	<0.001
	t	0.68	9.03		
	p	0.50	<0.001		

* p>0.05, Δp<0.05

### Comparative analysis of the incidence of adverse reactions between the two groups:

There were no new onset diabetic mellitus in two groups during treatment. The incidence of adverse reactions such as leukopenia, gastrointestinal reaction, fever and abnormal liver function were compared and analyzed between the two groups. The incidence of adverse reactions in the experimental group was 20%, significantly lower than that in the control group (42.5%), with a statistically significant difference (p=0.03). (Table-VI).

**Table VI:** Comparative analysis of adverse drug reactions between the two groups after treatment (χ¯±S) n=40.

Group	Leucopenia	Gastrointestinal reaction	Fever	Liver function damage	Incidence
Experimental group	3	2	1	2	8(20%)
Control group	6	5	2	4	17(42.5%)
χ^2^					4.71
P					0.03

P<0.05

## DISCUSSION

SLE can be associated with multiple viscera involvement, with rapid progression and easy recurrence, in which lupus nephritis (LN) is the most common one.^15^ Clinically, hormone combined with immunosuppressive agents is the preferred treatment for LN, with the main therapeutic purpose of protecting renal function, delaying the development of the disease and improving the long-term survival rate. A variety of drugs, including cyclophosphamide, cyclosporine, and mycophenolate mofetil, have been used clinically for the treatment of LN.^16^ Despite the clinical symptoms of patients can be improved to a certain extent by virtue of the above drugs, there are still deficiencies in efficacy and safety:^17^ complications such as bone marrow suppression and infection are prone to cause, with certain medication limitations.

Glucocorticoids are touted to have an effective clinical effect of inhibiting the synthesis of arachiidonic acid, leukotriene and other inflammatory mediators, reducing the release of inflammatory factors and activating the complement system.^18^ However, for LN with more abnormal immune link, the ideal effect is difficult to be achieved by a single target drug. Therefore, glucocorticoid therapy should be combined with immunosuppressive therapy to more effectively reduce organ damage caused by immune system disorders. Cyclophosphamide combined with glucocorticoids^19^ is a first-line drug for the treatment of LN, which inhibits specific antigens to stimulate the complement activity and transformation ability of lymphocytes, so as to block the development of kidney disease and achieve control of lupus activity.^20^ However, long-term use of cyclophosphamide will cause adverse reactions such as marrow suppression due to cytotoxic effects, and LN may recur after withdrawal.^21^

In recent years, the use of immunosuppressive agents for multi-target combination therapy has become a new method of clinical treatment of LN. Tacrolimus (TAC), a fermentation product isolated from Streptomyces, is a macrolide antibiotic and a powerful new immunosuppressor that can inhibit cellular immunity mainly by inhibiting the release of interleukin-2 (IL-2).^22^ It is usually used to prevent the rejection of kidney and liver transplantation.^23^ It is found in a study carried out by Mok et al.^24^ that the combination of TAC and prednisolone is not inferior to mycophenolate mofetil, and can be used for the treatment of active LN. After continuous administration for five years, no obvious nephrotoxicity and decreased renal function can be observed. In our study, the total effective rate of the experimental group was 92.5%, which was significantly better than the 75% of the control group (p=0.03); The indicators of the experimental group such as 24h urine protein quantification, serum albumin, serum creatinine, and urea nitrogen were improved compared with the control group, with statistically significant differences (p=0.00). Which were similar to the results of previous studies. It is reported in a 10-year RCT study that tacrolimus was significantly effective in the treatment of SLE and is expected to be a suitable target inhibitor for the treatment of LN.^25^ with few side effects. In this study, the incidence of adverse reactions was 20% in the experimental group and 42.5% in the control group, with a statistically significant difference (p=0.03), which can be supported by the conclusions of previous studies. According to the study of Zhou et al.^26^, TAC is effective and safe in patients with lupus nephritis. In addition, TAC boasts significant anti-inflammatory cytokine effects, including targeting IL-10 and transforming growth factor β, vascular endothelial growth factor, and tumor necrosis factor -α^27^ In this way, further damage to LN by inflammatory cytokines and inflammatory responses can be reduced.^25^ It has a certain synergistic effect with prednisone.^22^ While in our study, the levels of CRP, IL-6 and other inflammatory factors after treatment in the experimental group were lower than those in the control group, with a statistical significance (p<0.05). In addition, ESR, anti-DSDNA antibody positive rate and SLEDAI score were decreased compared with the control group, while complement C3 and C4 levels were significantly increased (p<0.05). Which were similar to the results of previous studies.

### Limitations of the study:

Few patients completed the clinical work of pathological examination; LN is a chronic disease with a long course, but follow-up was performed for a short time. In respond to this, active and effective countermeasures will be taken in the future clinical work to further extend the follow-up time, so as to more objectively evaluate the long-term benefits of this treatment regimen for patients.

## CONCLUSION

Tacrolimus combined with glucocorticoids is a safe and effective treatment regimen for patients with lupus nephritis, boasting a variety of benefits, such as significant efficacy and fewer adverse reactions. With such a regimen, the level of inflammatory factors can be significantly reduced, renal function indicators can be ameliorated, the patient’s ESR, complement C3, C4, anti-dsDNA antibody positive rate and SLEDAI score can be significantly improved.

### Authors’ Contributions:

**LL & YD:** Designed this study and prepared this manuscript, and are responsible and accountable for the accuracy or integrity of the work.

**YG & XQS:** Collected and analyzed clinical data.

**JJ:** Significantly revised this manuscript.
